# Describing practices of priority setting and resource allocation in publicly funded health care systems of high-income countries

**DOI:** 10.1186/s12913-021-06078-z

**Published:** 2021-01-27

**Authors:** Brayan V. Seixas, Dean A. Regier, Stirling Bryan, Craig Mitton

**Affiliations:** 1 Department of Health Policy and Management, Fielding School of Public Health, University of California, Los Angeles (UCLA), USA; 2 Cancer Control Research, BC Cancer and the Canadian Centre for Applied Research in Cancer Control (ARCC), Vancouver, Canada; 3 School of Population and Public Health, University of British Columbia (UBC), Vancouver, Canada; 4 Centre for Clinical Epidemiology and Evaluation, Vancouver Coastal Health Research Institute, Vancouver, Canada

**Keywords:** Priority setting, Resource allocation, Decision-making practices, Efficiency, Rationing

## Abstract

**Background:**

Healthcare spending has grown over the last decades in all developed countries. Making hard choices for investments in a rational, evidence-informed, systematic, transparent and legitimate manner constitutes an important objective. Yet, most scientific work in this area has focused on developing/improving prescriptive approaches for decision making and presenting case studies. The present work aimed to describe existing practices of priority setting and resource allocation (PSRA) within the context of publicly funded health care systems of high-income countries and inform areas for further improvement and research.

**Methods:**

An online qualitative survey, developed from a theoretical framework, was administered with decision-makers and academics from 18 countries. 450 individuals were invited and 58 participated (13% of response rate).

**Results:**

We found evidence that resource allocation is still largely carried out based on historical patterns and through ad hoc decisions, despite the widely held understanding that decisions should be based on multiple explicit criteria. Health technology assessment (HTA) was the tool most commonly indicated by respondents as a formal priority setting strategy. Several approaches were reported to have been used, with special emphasis on Program Budgeting and Marginal Analysis (PBMA), but limited evidence exists on their evaluation and routine use. Disinvestment frameworks are still very rare. There is increasing convergence on the use of multiple types of evidence to judge the value of investment options.

**Conclusions:**

Efforts to establish formal and explicit processes and rationales for decision-making in priority setting and resource allocation have been still rare outside the HTA realm. Our work indicates the need of development/improvement of decision-making frameworks in PSRA that: 1) have well-defined steps; 2) are based on multiple criteria; 3) are capable of assessing the opportunity costs involved; 4) focus on achieving higher value and not just on adoption; 5) engage involved stakeholders and the general public; 6) make good use and appraisal of all evidence available; and 6) emphasize transparency, legitimacy, and fairness.

**Supplementary Information:**

The online version contains supplementary material available at 10.1186/s12913-021-06078-z.

## Background

No matter how wealthy or large an organization, its available resources can never be deemed unlimited, because demand for new investments can grow indefinitely. Thus, organizations (and individuals) must constantly give preference to some types of spending over others. Individuals, companies, and governments alike are constantly selecting the priorities to which their scarce resources will be allocated. The process of assigning precedence to certain areas or services to receive investments is referred to in the scientific literature as priority setting and resource allocation (PSRA) or simply priority setting [1].

A scenario of ever-increasing health care spending and demographic and technological transformations have kept health care systems under budgetary pressures, posing enormous challenges for policy makers and health researchers. Thus, developing tools and knowledge to allocate scarce resources in the most efficient manner has become increasingly relevant in the field of health economics and public policy.

The scientific literature offers a rich view of a multiplicity of settings where formal frameworks of Priority Setting and Resource Allocation (PSRA) have been deployed. A recent scoping review [2] of priority setting practices in high-income countries identified 23 studies depicting specific practices in ten countries: Australia [3–5] Austria [6]; Canada [7–13]; Israel [14]; Korea [15, 16]; New Zealand [17]; Norway [18]; Sweden [19, 20]; UK [21–26]; US [27]. Among these studies, eight used Program Budgeting and Marginal Analysis (PBMA), eleven described a formal process that explicitly used a criterion-based approach to value assessment (with two of these using the ethics approach called Accountability for Reasonableness – A4R), three could be described as Health Technology Assessment (HTA) initiatives within a broader framework for decision making, and one was a modeling exercise using multiple criteria.

A small number of other existing literature reviews helps to provide a picture of scientific production in decision-making practices of priority setting and resource allocation. For instance, Polisena et al. [28] conducted a review of PSRA practices focusing on disinvestment, Barasa et al. [29] had a particular emphasis on hospital settings and Cromwell et al. [30] had a special interest in empirical frameworks with clearly defined guiding criteria.

The emerging narrative synthesis indicates that the majority of PSRA studies have been circumscribed in the context of high-income countries, despite a few important initiatives being studied and proposed elsewhere, as indicated by reviews conducted by Hipgrave [31] and Wiseman [32] emphasizing low- and middle-income settings. Even within the industrialized world, developing and implementing formal and explicit approaches for PSRA decision making has not received the same level of attention across countries. The papers found in the literature usually depict case studies of specific scenarios where a given methodology was tested, but usually not reported as formally evaluated or implemented in routine practice.

Whereas PSRA studies abound in countries such as the UK, Canada, and Australia, few or none are found from other high-income countries, at least in English-language journals. To our knowledge, there is no published study aiming to map existing processes of priority setting in a systematic manner across countries. Despite this, politicians, managers, and health professionals in all countries are determining priorities for investing scarce resources, even when no formal or explicit process is used. And in all decisions, some sort of rationale is necessarily implied, whether historical allocation, needs assessment, or any other non-expressed justification basis.

There is a gap *in the literature* between what is known and what is practiced. On what basis do hospitals allocate fixed budgets across internal sectors? How do health authorities decide to channel resources for a new capital investment? What rationale do state, provincial, or national governments employ to fund a new public health program or to cut an existing one? Are there formal, explicit, and transparent strategies underpinning such decisions in health care systems? Which stakeholders are normally involved? What types of evidence are used to inform decision making? Although some of these answers in respect to some countries can be found in the literature, there is no data whatsoever—and certainly no single source—for the majority.

The present work seeks to address this gap. Our overall objective is to describe existing processes of PSRA in publicly funded health care systems of selected high-income countries using an online qualitative survey with decision makers and researchers using Sandelowski’s theoretical framework of qualitative descriptive approach [33]. We have developed a rigorous and extensive reflection on the theoretical foundations for this study design and that has been published elsewhere. [34]

This study does not aim to provide a comprehensive mapping of PSRA strategies in any given context. The target is to generate more evidence on how decisions are commonly made across different levels of governance and administration (national, state/provincial, health authority, and hospital) in publicly funded health care systems in high-income countries. Rather than building a comprehensive map of existing practices, we aim to craft a mosaic of current procedures and rationales. Moreover, the study has the following specific objectives: 1) to provide a detailed report of common PSRA strategies across countries; 2) to describe the specific frameworks uncovered through the survey (both those previously unveiled and those known but not systematically documented); 3) to compare processes identified in the survey; and 4) to understand the common barriers and facilitators for the implementation of formal processes in the view of participants.

## Methods

### Research design

We employed an online qualitative survey [34, 35] with stakeholders that currently or previously worked with priority setting for publicly funded healthcare systems in high income counties. We intended to describe any area of PSRA that the respondent could have been involved with at any level of governance or administration (micro, meso, or macro) and type of health care (primary, specialized, hospital, etc.). We had to be aware that distinct depictions from different countries would arise simply because this is not a comprehensive work and those participating have different levels of interaction with the system. The study design is detailed below according to its three main components: sampling, data collection, and data analysis. Ethics approval for this study was secured from the University of British Columbia Behavioural Research Ethics Board – Certificate # H17–02009.

#### Sampling

Understanding a given phenomenon across different countries requires a minimum degree of comparability among the settings. Thus, the first choice was to focus on publicly funded health care systems, which broadly speaking could refer to systems or system components as diverse as the Canadian public single-payer insurance, the American programs Medicare and Medicaid, the British National Health Service (NHS), or the public component of social insurance systems like those in France or Germany.

**Fig. 1:**
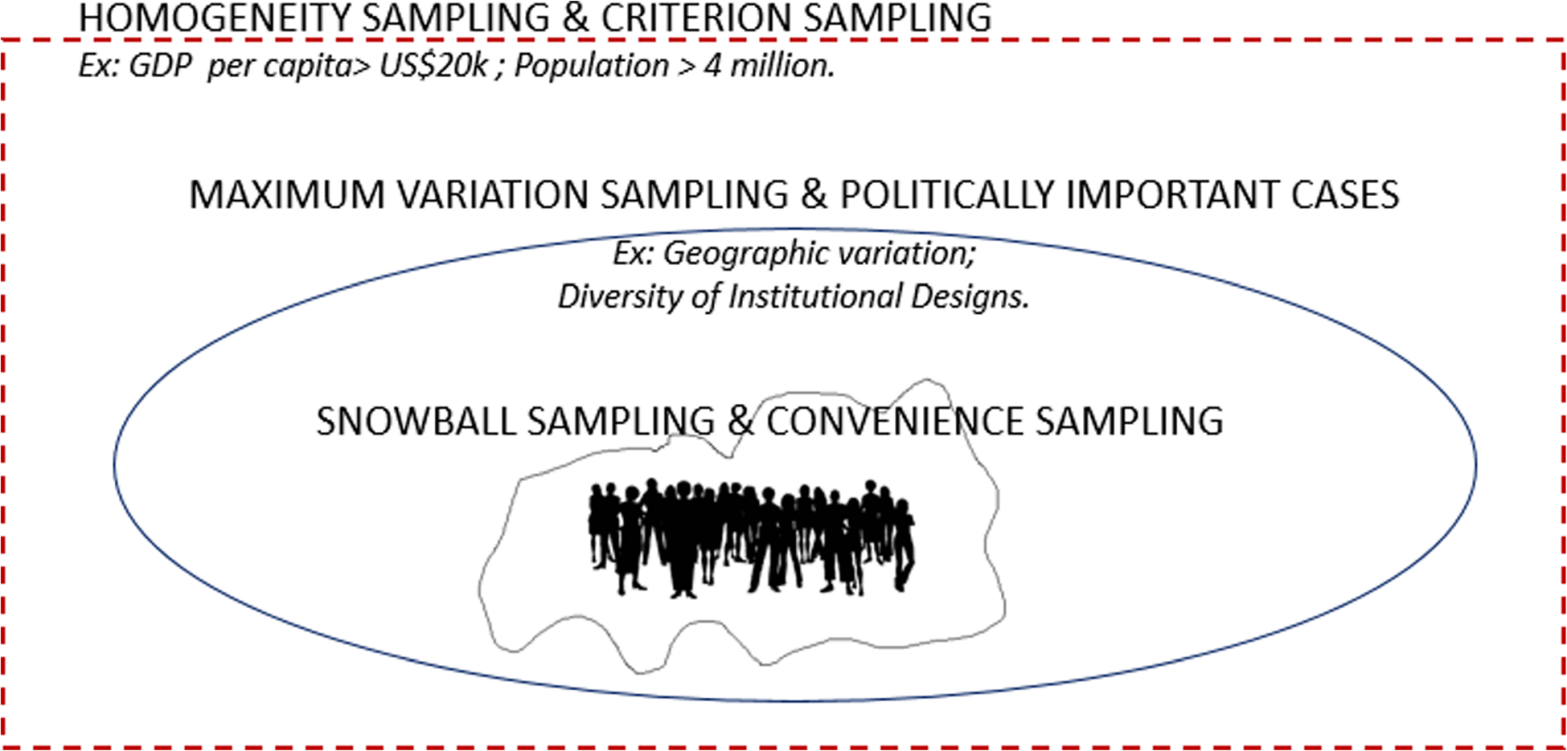
Purposeful sampling strategies for qualitative description in international comparative studies. The figure was created by the first author and has been previously published at Seixas et al., 2017. [33]

Following, it was necessary to decide which countries could potentially be included in the study. In order to achieve a minimum of comparability, two criteria were defined: countries with a population above 4 million people, and GDP per capita above USD 20,000 (2016 real dollars). These criteria serve the sole purpose of ensuring some comparability among the countries included in the study, not to identify potential participating countries from a formal classification system. This step corresponds to the highest level of sampling indicated in Fig. 1, encompassing strategies of purposeful sampling such as homogeneity and criteria sampling. Appendix 1 lists the 27 countries that meet these two criteria, according to data provided by the World Bank for the year 2016.

This list represents not all the countries included in the study, but rather the initial pool from where our sample could be obtained. Studying all 27 countries in the list would not be feasible, not only because of the overwhelming amount of data to be generated and analyzed but also because of the political nature of some of these places, like Kuwait and Saudi Arabia. To determine which countries from the pool would be included in our study, we applied other layers of purposeful sampling strategies described by Patton [36], like “politically important cases” or “maximum variation sampling”. See Seixas et al. (2017, 34) for a complete discussion on this methodological strategy. Table 1 shows the final list of the considered countries from which to draw respondents.

**Table 1:** List of countries included in the study

Countries		
Australia	Germany	Spain
Austria	Italy	Sweden
Belgium	Japan	Switzerland
Canada	Netherlands	United Kingdom
Denmark	New Zealand	United States
Finland	Norway	
France	Portugal	

The next step was to define and pursue strategies to find potential participants in each of the relevant countries to be included in the study. Two mechanisms were used to find out names to be invited to answer our survey.

1. We searched the scientific literature using Medline and PubMed, and we scanned the grey literature by conducting searches in websites of relevant organizations such as the International Society for Pharmacoeconomics and Outcomes Research (ISPOR) and the International Health Economics Association (IHEA). A complete list of sources used for this purpose is presented in Appendix 2. The search strategy consisted of combining a term related to PSRA and the name of a country, for instance, “priority setting” and “Finland”, “rationing” and “Finland”, or “resource allocation” and “New Zealand”. The terms used were either “priority setting”, “rationing” or “resource allocation” plus a country name. This was done for all countries in PubMed and Medline. All authors of works related to PSRA were recorded and included in our sampling list. The search results were pursued until saturation was achieved; whenever the same authors surfaced repeatedly, the search was stopped.
2. We searched the membership and contact list kept by the International Society on Priorities in Health (ISPH), provided by its management committee to find contact information of individuals working in any of the 18 countries included in our study.

All participants were also asked to suggest other potential participants. This snowball sampling was particularly important to reach out to decision makers who usually do not publish papers or are active members of those societies of health economics and related sciences. Further, no specific care setting was predetermined. Participants with experience and knowledge in all possible realms of health care (community care, pharmaceuticals, mental health, etc.) and all levels of governance and administration (national, regional, hospital, residential care, etc.) were considered.

#### Data collection

A survey instrument was developed and operationalized through the Qualtrics platform. A pilot study was run with four researchers, whose feedback led to improvements in the last version of the survey instrument. Participants were informed that the survey questionnaire (Additional file 2) could be answered either online through the Qualtrics platform or through phone/Skype calls, according to their preference.

#### Data analysis

As argued by Seixas et al. [34] and Sandelowsi [33], the analytical framework employed was qualitative content analysis. In addition, we developed a hierarchical analytical structure. The first underlying analytical layer consisted of a directed content analysis around structuring elements of PSRA decision-making practices (such as disinvestment, public engagement, use of evidence to inform decision making, etc.). These themes were chosen based on previous works from the literature [1, 7, 30]. These preconceived notions and categories were used to structure the survey questionnaire and ensure that participants’ answers would somehow address these elements. Some probing questions were also used to provide contextual information.

The second analytical layer consisted of the conventional content analysis [37], i.e., no previous system of codes was prepared beforehand to analyze each answer. The codes relevant for analysis emerged from the data. No information provided by participants was disregarded as irrelevant or impertinent. The typical process of assembling related codes into categories took a different format in the present work. Because participants refer to distinct scenarios and the primary purpose of our work is to describe existing practices as unique phenomena, coalescing different codes into the same category occurred only with participant data that referred to the same institutional context. Thus, data saturation was only sought and achieved (as it could possibly be in this research design) within a given specific scenario being referred by multiple participants. It did not prevent us, however, from comparing these categories across countries. The dataset was exported from the Qualtrics platform in a CSV file and analyzed using NVivo.

## Results

**Fig. 2:**
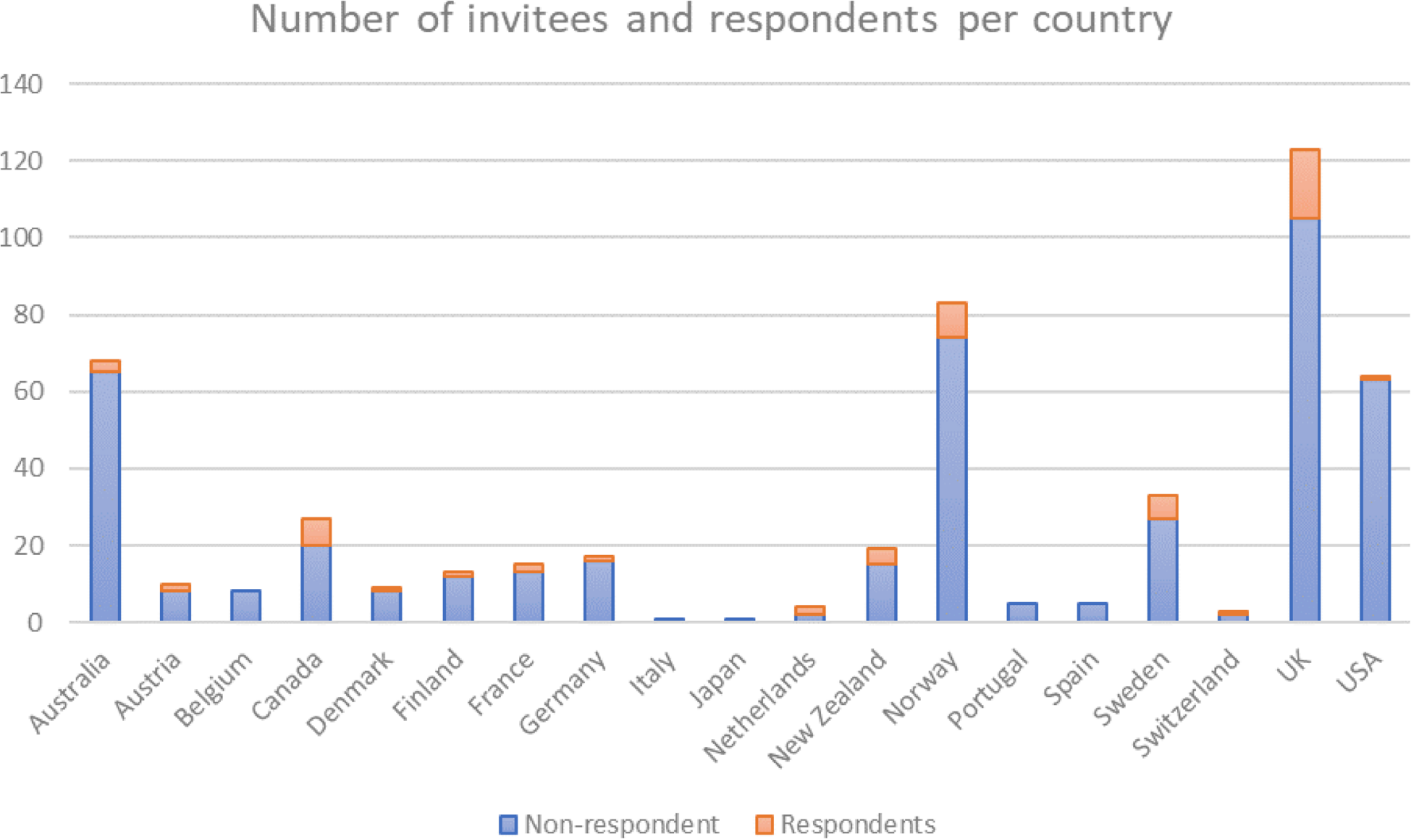
Number of invitees and respondents per country

In total, 450 individuals were invited to participate in our study from December 2017 and May 2018: 135 were identified in our initial search process, 44 were suggested through snowball sampling, and 273 contacts were obtained through the ISPH list. The list of invitees comprised people from all 18 countries included in the study. Fifty-eight individuals responded to the survey, yielding a response rate of 13%. Figure 2 shows the number of invitees and respondents by country. And Table 2 shows the primary professional role of those involved in the study, noting the number of academics/ researchers to decision-makers was approximately 6:1.

**Table 2:** Participants according to their country and primary professional role

	Academics / Researchers	Decision-makers
Australia	3	–
Austria	2	–
Canada	5	2
Denmark	1	–
Finland	1	–
France	2	–
Germany	1	–
Netherlands	2	–
New Zealand	3	1
Norway	8	1
Sweden	5	1
Switzerland	1	–
United Kingdom*	15	3
United States	1	–

**Fig. 3:**
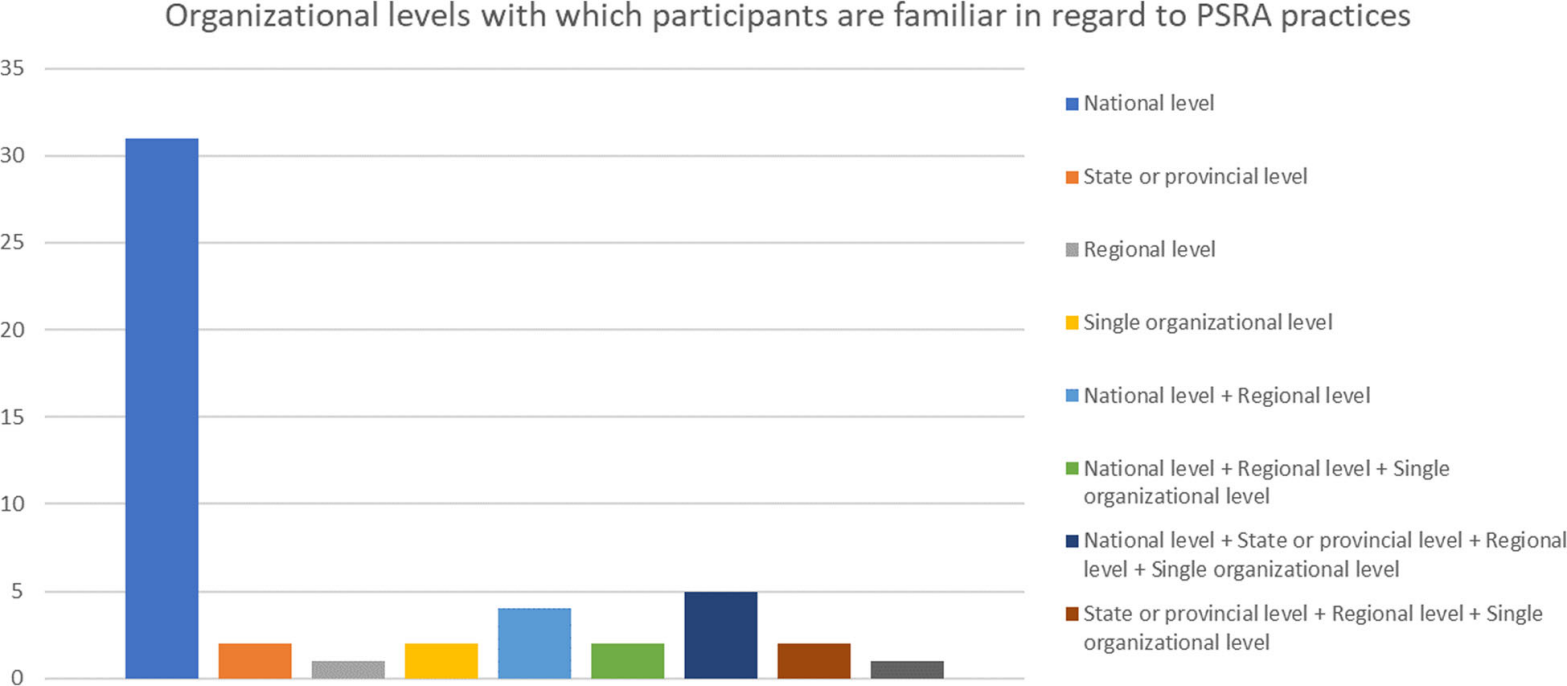
Institutional contexts with/where which participants work or have worked

Three participants (from Canada, Norway, and the USA) did not provide enough information to serve as an input for data analysis, and one participant from the UK answered the survey on the basis of priority setting for health research and not for resource allocation in managing health care systems. Thus, the data analysis was conducted with 54 participants. Of this number, 29 participants answered the survey focusing solely on practices of decision making in PSRA happening at the national level of their respective countries, 10 participants provided a brief overview of PSRA strategies across different levels of governance in their countries, and the remaining 15 either provided data about a specific setting (hospital, health authority, etc.) or provided generic information about practices that generally take place in hospitals, counties, and health authorities. As Fig. 3 shows, the majority of participants have experience or knowledge with practices of priority setting taken at the national level in their respective countries.

The findings are presented here according to the following topics: 1) PSRA frameworks at the national level; 2) PSRA frameworks at subnational entities and single health care facilities; 3) Disinvestment; 4) Use of evidence and appeals mechanism; 5) Stakeholder involvement and public engagement; 6) Facilitators and barriers for implementing formal PSRA processes; 7) Strengths of existing practices and areas for improvement.

### PSRA frameworks at the National Level

The participants who spoke about practices of priority setting taking place at the national level of their respective countries mostly described activities of economic evaluation of health care technologies carried out by HTA agencies or the like. For example, respondents from Australia reported the work of the Pharmaceutical Benefits Advisory Committee (PBAC) and the Medical Services Advisory Committee (MSAC), while a Canadian participant described the economic evaluation process promoted by the Canadian Agency for Drugs and Technologies in Health (CADTH). A complete list of PSRA frameworks described by participants is found in supplementary Table 1.

Participants from Norway and Sweden focused on the national legislative platforms on priority setting. Parliaments from both countries have passed legislation that sets general principles and notions for priority setting in health care. In Norway, decision-makers must base investment choices on the basis of three criteria: benefit, resource consumption (or budget impact), and disease severity. In Sweden, the ethical platform of PSRA has three guiding principles: human dignity, need/solidarity, and cost-effectiveness. Interview participants reported that these national policies do not explain procedures for arriving at decisions and leave lots of room for interpretation (regarding outcomes measurement, threshold, procedures, etc.)

In Finland, the PSRA framework practiced by The Council for Choices in Health Care (COHERE Finland) was identified. According to a respondent, the multidisciplinary council relies on the following three principles to make decisions: “significance of a health issue ( …), medical justifiability ( …), and ethical and financial aspects as a whole ( …)” . It was not clear how decisions are reached by the council or how evidence is collected and appraised.

Dutch respondents focused on three processes at the national level in the Netherlands. One is a program run by the National Health Care Institute (Zorginstituut Nederland [ZIN]), called Zinnige Zorg [Appropriate Care], which conducts systematic analyses per ICD-10 chapter to ensure patient-oriented, effective, and medically necessary care. Furthermore, ZIN has an appraisal committee related to HTA that relies not only on cost-effectiveness but also on criteria like budget impact and added therapeutic value. One participant from the Netherlands also reported the use of a formal process using a value assessment framework based on Multi-Criteria Decision Analysis (MCDA) and A4R.

At the national level, most UK participants described the work of the National Institute for Health and Care Excellence (NICE). The converging narrative is briefly summarized by the following sentences from a British participant: “So, there is indeed a formal process, and the rationale provided by NICE for it is essentially about the need to balance a pseudo-utilitarian concern for the maximization of aggregated population health and what they conceive of as egalitarian concerns, which are focused on who receives the benefits. The resulting process is built around a soft incremental cost-effectiveness threshold set at £20000-£30000/QALY, which is balanced against a set of counterweights (severity, end-of-life premium…).” Apart from the NICE activities, one participant also mentioned the work of the “Advisory Committee on Resource Allocation that allocates funds geographically according to two criteria: offering equal access to care for equal need; and reducing avoidable variations in health inequalities”.

### PSRA frameworks at subnational entities or single health care organizations

A mosaic of the most nuanced findings from different subnational contexts or single organizations is discussed in this section. Supplementary Table 1 provides a thorough overview of our findings.

An Austrian participant reported that some hospitals have set up committees of clinicians for priority setting: the rules are based on benefit assessment (gains in quality of life and years of life) and budget impact. The basic underlying principle is utilitarian thinking of maximizing health. No further detail regarding the extent to which the committees have been implemented, evaluation of current practices, or even the exact procedures to make decisions was provided.

We collected two sets of data about processes taking place at non-national levels of Canada. One included reporting from four respondents about the PSRA process taking place at one provincial level institution. The other consisted of a general report about practices in different settings provided by an academic with extensive experience providing guidance on PSRA to health organizations. This particular provincial institution has a formal process carried out by an internal committee to evaluate and prioritize coverage of drugs and non-drug technologies for cancer care. The committee evaluates submissions on the basis of clinical effectiveness, although some level of discussion around cost-effectiveness and budget impact occurs without formal influence in the final decision. A list of prioritized drugs is then sent to the executive committee, who make final recommendations to the provincial Ministry of Health. A participant said that “in the past they used a mix of clinical and cost-effectiveness for decision making, but the way in which the criteria were applied was confusing and not systematic”.

Another Canadian participant reported the implementation of an explicit values-based decision-making framework informed by accountability for reasonableness and program budgeting and marginal analysis (A4R and PBMA) [38, 39] in “a variety of organizational settings (e.g., provincial ministry, large hospitals, local health integration networks) and organizational levels (e.g., ministry emergency operations centre vs. deputy minister’s office; ICU vs. organization-wide clinical priority setting)” across Canada. According to the participant, most of these organizations are fairly sophisticated and have formal decision-making processes in place and have requested consultancy to “enhance transparency and public defensibility of the priority-setting process, to balance competing goals, values, and interests in a rigorous way, and/or to strengthen effective engagement of affected stakeholders”.

Two national leaders in the field and staff members of the National Centre for Priority Setting in Health Care that answered the survey together provided an insightful overview at the sub-national level with respect to Sweden: “All the 21 regional health authorities (county councils/regions) in Sweden have a formal process for the setting of annual budgets. This is of course an example of resource allocation, but it is hardly a process where formal priority setting is done (a structured process for ranking of interventions). How are priorities for investments established on the regional level in Sweden? This differs—initiatives that will determine priorities can emanate from regional politicians and political parties, from local clinical leaders, the interpretations of national guidelines issued by the state authority National Board of Health and Welfare (Socialstyrelsen).” This general view is confirmed by another Swedish participant, who stated that “on local or clinical level there is no unifying priority [setting] process”.

The data obtained from UK participants may appear controversial but seems to reflect an environment with varying levels of capacity to deal with priority setting across health organizations. Two participating academics with expertise in PSRA reported that most local and regional organizations do not have a formal, explicit, and systematic process. In the words of a third British participant, “most decisions are taken in a fairly ad hoc manner locally or regionally, despite a great deal of thought academically about the right way to undertake priority-setting processes”. Nonetheless, other participants have provided insights on formal processes taking place in some health organizations. At the local/regional level, the Clinical Commissioning Groups (CCG) are the main structures responsible for priority setting. Although some level of guidance is established by national authorities (like NICE), the CCGs need to make their own population spending decisions. One reported that, based on his experience assisting several CCGs in this matter, “Many have a “prioritisation” process … and that these will typically incorporate some fairly rough and ready version of MCDA. Budgets are extremely tight and so many prioritisation policies ask that the budget for any proposed investment be identified before the investment options will be considered. The criteria often found in these policies include: strength of clinical effect, strength of evidence, availability of alternatives, fit with national and other guidance (e.g., local sustainability and transformation plans), and cost-effectiveness. Other criteria used less frequently or with less weighting include: patient and public consultation/affordability and implementation issues/legal risks.”

### Disinvestment

In most places, respondents were not aware of any formal and systematic approach for disinvestment. Nevertheless, some participants commented how the concept of disinvestment may manifest in their national context. A Dutch respondent highlighted the existence of a program related to “Choosing Wisely”, used for prioritizing HTA research. A participant working in a Norwegian health organization said that although no formal procedures exist, decision makers discuss disinvestment in meetings twice a month considering that, for example, “investment in more advanced technologies may lead to task change for staff, ending up perhaps in reduced staff”.

In Canada at the national level, one respondent said that CADTH is in process of establishing a disinvestment framework. The participant further stated that “HTA by definition is comparative so every decision to invest in something should come with a decision to not invest in a comparator (an oversimplification I know but the intent is not to have two unwieldy disconnected processes)”. Participants at the Canadian provincial institution referred to earlier reported that there is no formal framework for disinvestment. Nonetheless, two respondents pointed out that disinvestment does take place naturally with incorporation of novel technologies. A participant with involvement in various health organizations within Canada stated that in their experience: “Disinvestment processes are less well developed than investment-based priority setting. It is usually for this reason that I am called in to provide advice, recognizing that disinvestment may often be experienced as a loss by affected stakeholders. Hence, there is a sense of urgency to ‘get the process right’ to establish the legitimacy and fairness of priority setting in the eyes of affected stakeholders. Population need and clinical effectiveness are emphasized, although evidence is often not robust or readily available.”

Participants in the UK state that there is no formal framework for disinvestment at the national level. They emphasized that “NICE tends to focus on assessing the cost-effectiveness of new entrants to the benefits package (drugs, new technologies)”. Some participants pointed out some NICE initiatives as “disinvestment decisions”, such as the “do not do” list (i.e., a compilation of hundreds of interventions deemed to be low value) and a “cost saving guidance” that includes orientation on the treatment of respiratory tract infection in primary care, which indicates that, apart from reducing antibiotic resistance and medicine related adverse events, the use of a “no prescribing” or “delayed prescribing” policy is expected to decrease antibiotic prescribing by a few million pounds nationally. One participant in particular, on the other hand, commented that the emphasis of NICE on adoption tends to push the whole system to focus on “what is new and coming from NICE” rather than on what is more practical and of greater value, but not subject to national appraisal.

Whereas NICE issues guidance and recommendations of technologies to be funded, CCGs in the UK are in fact the organizations ultimately responsible for disinvestment activities. The practices around it vary considerably across the country. Participants reported that CCGs have been going through a period of financial difficulty, leading to mandated savings targets from different service areas that are then expected to present savings proposals. Regarding this aspect of CCGs, one participant who has worked with various CCGs indicated that they seem to have been putting more emphasis on disinvestment than on priority setting, establishing separate procedures for conducting disinvestment. In his words: “they are a reaction to the relentless proposals for new investment”. Further details on the exact design of such “separate processes for disinvestments” were not provided.

### Use of evidence and appeals mechanism

When asked about the types of evidence used to inform decisions, 41 participants (72%) indicated that there is reliance on epidemiological, clinical, and economic evidence and expert opinions. But this does not mean that these pieces of information have equal or equivalent status. Several participants highlighted that expert opinions are only sought in the absence of “hard evidence”. Others pointed out that data on budget impact analysis or cost-effectiveness have less importance in their contexts when compared to clinical effectiveness.

Responses about the existence of appeals mechanisms indicate that there is no well-established understanding about what would constitute an appeals mechanism. 35 participants (60%) answered “no” or “uncertain” for the existence of such an instrument within their organizational or contextual setting. The other participants often provided information on processes that they were unsure could be deemed appeals mechanisms. Several participants indicated the legally entitled right to complain about health care services as the appeals mechanisms in their countries. Two participants from Norway stated that patients can file formal complaints about the services they are receiving or are supposed to receive.

Within the context of HTA, respondents from Austria, Australia, and the UK highlighted the fact that coverage decisions can be appealed in court. In Australia there may be a request of a technical review capacity of whether PBAC has made an error through an external expert review; or, as a respondent put it, after rejection, companies may resubmit the application to alter dosage or the price. In the United Kingdom, legal challenges of NICE decisions have occurred. According to participants, these can be the result of political pressure by the society, patient groups, or pharmaceutical industries.

### Stakeholder involvement and public engagement

Within the HTA realm, respondents reported a well-established culture of stakeholder involvement. Varying according to country-specific system designs, a wide variety of stakeholders has been included in the decision-making process, such as clinicians, managers, health economists, manufacturers, insurance companies, bureaucrats and politicians. Patient experience has been increasingly considered through a variety of different forms (consumer hearings, focus group, patient representative organizations, etc.)

Yet, methods for engaging the general public and assessing societal values and preferences are just appearing “in the radar” and are not common practice. Usually, the public is only involved insofar as there is space for consumer input throughout the process or suggesting topics for analysis. Respondents from Canada, Netherlands, and Sweden stated that several experiments with public consultation have been tried, but they have been circumscribed to research contexts. In Canada, for example, a health authority engaged its community advisory committees circa 2002 in developing priority-setting criteria and a hospital “created a citizen’s council to review evidence and make recommendations on health service investment/disinvestments to aid the hospital in addressing a budget deficit”. Dutch participants reported a project called “Burgerforum” (literally ‘civilian forum’), which aims to elicit public preferences for the HTA decision-making process.

With respect to decision-making practices at local/regional level in the UK (i.e., within the context of CCGs), one participant stated that there is often a stakeholder panel, mostly composed of the main budget holders and provider organizations affected by the decisions, including organizational leaders and senior clinicians. These stakeholders are either scoring options or reviewing a scoring exercise for various investment options. He points out that, “it is not always clear a) whether those participating are expected to shed organizational affiliations or b) how recommendations are to be ratified and put into practice. Some bodies will have formal decision processes (e.g., establishing how and when to vote) whereas others handle this much more informally and are prey to who turns up!” In regards to public engagement, one commented: “Despite lofty rhetoric the NHS is still resistant to public involvement, especially when it involves contentious decisions about limiting access to services. Consultation processes are sometimes undertaken but these are often highly generalized and aspirational in tone (i.e., ‘what do you think about our new vision for world class health care in your area?’). The nitty-gritty of decision making often involves a few worthy individuals who buy into the ‘volunteerist’ model—i.e., they are there to help the system first and foremost. Big priority-setting decisions are often then subject to opposition and challenge through campaigns, media etc.”

To this point, we have presented the qualitative findings with a predominantly factual nature, those directly related to the actual description of processes and practices in priority setting. In other words, the data where most people would readily agree upon, the proper matter of qualitative description as understood by Sandelowski [33]. The next two subsections, in turn, reveal findings with a more interpretative hue. They pertain to participants’ critical views of the process. Our analysis simply summarizes those findings, without theorizing them, keeping the analytical work at a fundamentally descriptive level.

### Facilitators and barriers

Participants were also asked to indicate which elements of their institutional or contextual contexts they identify as facilitators and barriers to developing and implementing formal and explicit processes of priority setting. A comprehensive list of the elements pointed out by participants per country is presented in Supplementary Table 2. For some countries, responses refer to the broad national context whereas a setting-specific analysis is provided for others. The most commonly cited facilitators are budgetary pressures, strong leadership, and existence of key champions. The most common barriers were lack of knowledge about PSRA, vested interests, political resistance, lack of trust among stakeholders, and media pressure. In addition, some elements suggested are specific to their context. For instance, in Norway the existence of a strong welfare system with emphasis on equity was pointed out as a facilitator because the society tends to better accept the prioritization need. Likewise, Australian participants state that the fragmented nature of their health system, leading to “too many players at too many levels”, is a barrier for the establishment of formal and explicit frameworks of decision making.

### Strengths and areas for improvement

We also asked survey participants about strengths and areas needing improvement in the existing practices of PSRA in their settings/regions/countries. The full set of findings per country is presented in Supplementary Table 3. In regard to the work carried out by HTA bodies in Australia, Canada, Netherlands, New Zealand, Norway and Canada, respondents pointed out as strengths: good level of expertise, the systematic and careful use of evidence to inform decisions and the explicitness of rationales guiding decisions. In relation to broader PSRA practices, respondents from Sweden and Norway argued that the existence of national platforms with legally defined principles, which confer political legitimacy for the process, represented a strong asset of their institutional practices of decision-making. When it comes to local organizations or subnational governance, respondents from the UK and Canada said that existing strengths are knowledge of local realities and stakeholder involvement.

The areas for improvement suggested by respondents were more numerous and diverse across settings, but a few converging topics were identified: capacity building (there is a need to better understanding of economic notions driving PSRA); a greater emphasis on disinvestment, instead of adoption of new technologies; consideration of social values / public engagement; and formal guidance on how to translate legal principles or theoretical knowledge into consistent decision-making processes.

## Discussion

The present work constitutes to our knowledge the first attempt to describe practices of priority setting and resource allocation across countries in a systematic way beyond sole reliance on papers and reports. In addition to filling a gap in the literature, this work also makes a contribution through use of a theoretically robust methodology in the field of international studies on public policy [34]. Further, and in our view most importantly, it offers a better understanding of current practices of decision-making in PSRA and, thus, provides insight for both researchers and decision-makers about the elements that need to be both further researched and addressed in policy-making contexts across countries.

In spite of several decades of work on PSRA in health care, we identified a gap in the literature regarding the mapping and documenting of existing practices of decision making in PSRA, often assumed to be limited in a given context to historical/political allocation or HTA. We further wanted to extend insight gained through review of published case studies often reporting on use of various decision analytic techniques. In short, our work intended to rely on a methodology that could offer a better understanding of the existing practices in priority setting. Although previous qualitative works has been conducted in the field of priority setting [8, 40–42], they have focused on single jurisdictions/organizations. Our work is unique in applying qualitative research to understand PSRA decision-making processes across different settings and countries.

Our findings showed that priority setting has received different levels of attention and action across different countries. Whereas scarcity has been perceived quite acutely in the UK, for example, a participant from Switzerland reported the perception that there is sufficient money in the system. In Norway the topic has been discussed since early 1980s, whereas in Austria it has not been an object of debate in the public sphere. Consequently, observed practices vary considerably from country to country and even within national contexts.

In Sweden and Norway, national guidelines of priority setting were approved by the parliament. The establishment of criteria and principles at the national level has been pointed out as a strategy to make decision-making processes accountable, fair, and systematic. Nonetheless, what was demonstrated in this work is that existing practices still vary considerably sub-nationally and there has not been a systematic and explicit process of making the actual decisions in priority setting. The national guidance has been viewed by some to be vague and of little practical value.

What is clearly detectable in the findings is that efforts to allocate resources efficiently and determine priorities are concentrated on *one-off decisions about adopting and reimbursing health care technologies*. The vast majority of efforts on HTA are directed to deciding whether to incorporate a new technology in the package of services covered or reimbursed. In this sense, we observe that HTA has an overemphasis on adoption of new technologies [43].

Many participants indicated that decision making processes in HTA are the de facto formal strategy of PSRA in their institutional setting. Given the nature of our qualitative descriptive study, we stayed close to the surface of the data, with the least possible level of interpretation. HTA activities were shown as existing practices of PSRA, following the participants’ perspectives. Yet, this is a particularly contentious view. We have argued elsewhere [44] that HTA is a tool for management of health care technologies that offers evidence to support decision making. It is a value assessment framework [45], i.e., it enables the appraisal of the value for money of a given technology in comparison to others, but it does not offer an answer on prioritization of investments broadly speaking. In reality, although the majority of participants did stick to this view of HTA as a tool of priority setting and resource allocation, two academics from the UK pointed out that HTA does not explicitly account for opportunity costs and is not a PSRA methodology in itself.

The idea of disinvestment in general was demonstrated to be only incipiently discussed within health care systems. Despite the lack of formal initiatives on disinvestment, the concept has become more important given the growing budgetary pressures on health care systems. Again in reference to the UK, it was suggested that at the regional level there is a focus on processes to disinvest from treatments and even entire services to stay within the established budget rather than focusing on developing practices of priority setting in a more systematic way. There is debate in the literature over whether a framework that does not consider disinvestment is really a framework of priority setting. While there are no clear answers on this in the current study, it is helpful to be reminded that we did not have a priori identified categories for defining priority setting.

In spite of the lack of formal approaches reported by respondents to determine priorities for investment beyond the HTA realm, observing the existing practices offered a rich view of certain phenomena. There seems to be wide agreement on the use of multiple criteria to inform decision making. The majority of formal approaches take more than a single criterion into consideration, and also suggest that certain criteria should receive more or less weight. This is the case of the Canadian provincial institution, for example, where decisions are made primarily on the basis of clinical effectiveness, although other elements tend to be raised in the discussions.

Another point of convergence is the appraisal of the available evidence. Almost all participants reported that all types of evidence are taken into consideration. This represents a shift from the traditional paradigm of “hard evidence” or “gold standard” status attributed to randomized controlled trials (RCT) to a more holistic understanding of health care systems. It does not mean, however, that all types of evidence are treated equally. Evidence of clinical effectiveness still enjoys more prestige and is deemed superior to others.

An aspect upon which there is no common understanding is appeals mechanisms which is seen as a key principle within A4R [46]. While some participants reported that there is appeal in some scenarios— individuals can consult another doctor for a “second opinion” or companies and patient groups can take decisions to court—other participants reported the total absence of appeals or even the debate about it within the same settings. The practices described in this work also do not tend to have formal structures and mechanisms to engage the general public. Notwithstanding the debate around eliciting and incorporating societal values in decision-making frameworks, almost all participants reported processes without an explicit concern in this realm.

A further related topic, and one where there is more commonality, is transparency. Whether mentioning it as a strength or an area for improvement, most participants emphasized the importance of transparent and explicit processes of decision making. And even in settings where legally defined guidance exists, such as Norway and Sweden, respondents commented that it is important to be fully transparent in respect to the exact procedures leading to the final decisions, i.e., the actors influencing it, the underlying rationale, the voting/rating system, etc.

The countries included in the study, despite belonging to a generic group of high-income countries, constitute very different societies. They differ in institutional design, e.g., the model of health care system (multi-payer or single-payer, social insurance or public service, etc.), political system (presidentialism or parliamentarism, counties or regions, etc.), infrastructure, economic dependence of certain industries, levels of social and economic inequality, and underlying values (about the role of the state and the size of markets, about equity, about an individual’s responsibilities for their own health, etc.). They represent a diversity of underpinning societal contexts upon which health care systems are created. This is reflected in the ways that certain needs are perceived, on the ways that problems are addressed, and the extent to which countries are affected by local and international issues. The methodology chosen was a survey designed within the realm of qualitative descriptive studies. Its underpinning theoretical reflection, discussed elsewhere [34], allowed us to work within the differences that were present in this diverse set of countries.

In many aspects, our work confirms findings obtained from existing systematic reviews, like the lack of formal evaluation of PSRA exercises and their implementation in routine practice [30] and the predominance of initiatives around adoption of new technologies, with rare approaches for disinvestment [28, 29]. In resonance with our findings about the generalized deficiency of clear guidance for translating theoretical knowledge and ethical/legal principles in consistent and routine decision-making, Hipgrave et al. [31] state that “the overarching conclusion was that even in high-income countries participator participatory, accountable and rational approaches to health priority-setting should be achievable, the process and outcomes of such exercises have been unsatisfactory” (p.192). But the present work also sheds light on matters not identified in the literature, like the wide understanding that decisions require multiple criteria (not only maximization of QALYs), the growing convergence in the reliance of multiple types of evidence to inform decisions (shifting from the idea of RCT as the gold standard evidence), and the need of a debate around appeals mechanisms. It is.

In terms of limitations, most critically, the methodology relies on the word of participants to depict existing institutional practices, which can be misleading. The respondents’ testimonials about the processes of decision making in PSRA tend to vary according to the level of “formal knowledge” (i.e., knowledge of what is formally established and documented) and experience with the setting. So, considering that all participants have good faith in responding to the questionnaire, the veracity or faithfulness of the answer may be compromised because of a biased or partial understanding of the actual practices. Obviously, describing a process based on reports of several individuals is desirable to provide a fuller representation of the actual process and to confront inconsistencies or discrepancies among answers. The information obtained for certain settings was particularly weak, given the participation of a single or a few respondents, as was the case for Denmark, Switzerland, Germany, and the USA, where only one individual completed the survey and an insufficient level of quality data was provided. In addition, the overall response rate was quite low and it is not unlikely that relevant initiatives have been still overlooked.

On the other hand, in the settings with multiple participants, the notion of data saturation was pertinent because the reading and categorization of a further participant would allow the confirmation of emerging themes. The convergence of topics and ideas was important to illuminate what is perceived as utterly important in each setting. Possible divergence was not used to weaken previously emerged themes, but rather it was used to expand the horizon of codes. At the predominantly descriptive process, divergence could mean disparate and equivocal reports of existing practices, but that did not occur in the present work. The answers did not provide controversial description of processes.

The use of snowball sampling as a strategy to reach out to decision-makers did not work very well and the final sample had a much higher number of academics. Yet, we did not observe any systematic difference between academics and decision-makers.

Another limitation is the restriction to high-income settings. The priority-setting scenarios described in this work may be different in low- and middle-income countries. It is possible that interesting PSRA practices have not been captured here simply for taking place elsewhere. Further research on other settings is needed.

The data analysis was based on a qualitative content analytical approach [37, 47], which focus on the factual content of the data. [48] Thus, the explicit value and moral judgements had a secondary role, to provide us more context. We presented the data and avoided making assumptions, generalizations, and deep interpretations. Instead, we allowed participants to speak in our analytical narrative, often quoting them verbatim. Some respondents provided references to compose their answers, which were also used to present a more detailed and faithful depiction of the institutional realities being discussed. Given the predominantly descriptive nature of this work, discussions around generalizability or transferability may not be appropriate.

Despite the focus on revealing the factual content of existing practices of PSRA, the survey instrument also asked participants about their views on some aspects of the system, such as elements in their settings that could work as facilitators or barriers for the implementation of explicit and formal PSRA processes, as well as strengths and areas for improvement of existing practices. This secondary objective of our work was important to better understand the underlying structures upon which decision-making frameworks are developed and implemented as well as the envisioned pathways for public policies and the research field. Questions 11 and 12 of the survey (Additional file 2) asked about the perceptions of fairness and the overall rating of current decision-making practices. However, the answers followed no identified pattern and were quite difficult to be analyzed in a meaningful way. Therefore, we simply presented the data without conducting any further analysis.

Overall, the methodology demonstrated an insightful and auspicious strategy for qualitative description of institutional aspects of public services in international studies. Nonetheless, identifying potential participants and engaging an adequate number of them presents a challenge. In our case, the strategy of sampling was not successful in reaching regional and local decision makers in the countries included. Despite this, we believe our study provides a useful mosaic of practices deemed to be PSRA processes and offers a good basis for a better grasp of how priority setting has been understood and translated into practice across many different countries.

## Conclusions

The provision of health care has consumed more and more resources in virtually all countries in the developed world. Demographic and technological changes have played a significant role in this phenomenon, increasing health cost per capita considerably. Recognizing its unsustainability has made researchers, decision makers, and politicians aware of the need to make choices on where to invest scarce resources. Although scarcity has always existed, it has recently achieved unprecedented levels in the modern history of wealthy nations.

Having this ongoing phenomenon in mind, we sought to investigate the practices of decision making in PSRA in health care systems in high-income countries. The intent was not to obtain a complete map or comprehensive database of existing strategies but rather to produce a mosaic or “photo album” of current practices.

Our findings can be briefly summarized as follows. First, resource allocation in health care has largely been based on historical patterns and through ad hoc decisions. Second, according to the majority of participants, the most commonly employed PSRA strategy is HTA. Nonetheless, as it was argued by some participants, this is a theoretically controversial statement given that HTA would not constitute per se an actual tool to elect priorities and allocate resources given that it does not properly take into account the opportunity costs across different areas of investment. Third, Disinvestment frameworks are very rare and the topic itself has only just begun to appear with any regularity, but yet its importance is clear.

Fourth, several initiatives of PSRA have been tested and published, with special emphasis on PBMA, but there is limited evidence that health organizations have continuously used them in a systematic way across countries. Next, countries that have established a national policy on priority setting, indicating principles or criteria to base decisions on, have not provided pragmatic guidance to decision makers who still want advice on practical procedures to allocate resources efficiently and fairly.

There is growing understanding that decisions must be based on multiple criteria. Yet, the MCDA approach has been applied variably. Another point of increasing convergence is the reliance of multiple types of evidence to judge the value of investment options, perhaps identifying pragmatism as a leading principle in PSRA.

Public engagement is not a reality in most places. Decision makers and researchers at points do not know how and when to involve the general public in the decision-making processes. There seems to be an absence of debate about what would constitute an appeals mechanism at a practical level within this context.

Our findings suggest an agenda for the research field on PSRA, which includes the development/improvement of decision-making frameworks that: 1) have well-defined steps; 2) are based on multiple criteria; 3) are capable of assessing the opportunity costs involved; 4) focus on achieving higher value and not just on adoption; 5) engage involved stakeholders and the general public; 6) make good use and appraisal of all evidence available; and 6) emphasize transparency, legitimacy, and fairness.

### Supplementary Information

**Additional file 1.**

**Additional file 2.** Questionnaire

## Data Availability

Anonymized data available upon request through emailing brayanseixas@ucla.edu.

## Appendix 1

### Initial pool of countries to be sampled

**Table:** Table 3

Country	GDP per capita (2016 USD)	Population
Australia	49,755.32	24,210,809
Austria	44,757.63	8,731,471
Belgium	41,271.48	11,338,476
Canada	42,348.95	36,264,604
Denmark	53,578.76	5,728,010
Finland	43,433.03	5,495,303
France	36,857.12	66,892,205
Germany	42,161.32	82,487,842
Hong Kong SAR, China	43,740.99	7,336,600
Ireland	64,175.44	4,749,777
Israel	37,180.53	8,546,000
Italy	30,668.98	60,627,498
Japan	38,972.34	126,994,511
Korea, Rep.	27,538.81	51,245,707
Kuwait	27,359.23	4,052,584
Netherlands	45,637.89	17,030,314
New Zealand	39,412.49	4,693,200
Norway	70,867.94	5,236,151
Portugal	19,871.72	10,325,452
Saudi Arabia	20,028.65	32,275,687
Singapore	52,962.49	5,607,283
Spain	26,616.49	46,484,533
Sweden	51,844.76	9,923,085
Switzerland	79,887.52	8,372,413
United Arab Emirates	37,622.21	9,269,612
United Kingdom	40,412.03	65,595,565
United States	57,638.16	323,127,513

## Appendix 2

### List of Organizations’ Websites Used in the Grey Literature Search to Identify Potential Participants

**Table:** Table 4

Organization	Official Website Address
MSAC	www.msac.gov.au
HealthPACT	https://www.health.qld.gov.au/healthpact/
PBAC	https://pbac.pbs.gov.au/
Norwegian Council for Quality Improvement and Priority Setting in Health Care	http://www.prioritering.no/
SHTG	http://www.healthcareimprovementscotland.org/
Osteba	http://www.osakidetza.euskadi.eus/
Avalia-T	http://www.sergas.es
SBU	http://www.sbu.se
NICE	www.nice.org.uk
HTAi	www.htai.org
INAHTA	htttp://www.inahta.org
ISPOR	www.ispor.org
EUnetHTA	http://www.eunethta.eu
IHEA	www.healtheconomics.org
AHRQ	http://www.ahrq.gov/
CADTH	www.cadth.org

## Abbreviations

A4R: Accountability for Reasonableness
AHRQ: Agency for Health Research and Quality
CADTH: Canadian Agency for Drugs and Technologies in Health
CCG: Clinical Commissioning Groups
CEA: Cost-Effectiveness Analysis
COHERE: The Council for Choices in Health Care (Finland)
CUA: Cost-Utility Analysis
EUnetHTA: European Network for Health Technology Assessment
G-BA: Gemeinsamen Bundes-Ausschuss [The Federal Joint Committee] (Germany)
GDP: Gross Domestic Product
GKV: Gesetzliche Krankenversicherung [The statutory health insurance system] (Germany)
HTA: Health Technology Assessment
HTAi: Health Technology Assessment International
IHEA: International Health Economics Association
INAHTA: International Network of Agencies for Health Technology Assessment
ISPH: International Society on Priorities in Health
ISPOR: International Society for Pharmacoeconomics and Outcomes Research
MCDA: Multi-Criteria Decision Analysis
MSAC: Medical Services Advisory Committee
NHS: National Health Service (United Kingdom)
NICE: National Institute for Health and Care Excellence
PBAC: Pharmaceutical Benefits Advisory Committee
PBMA: Program Budgeting and Marginal Analysis
PSRA: Priority Setting and Resource Allocation
QALY: Quality-Adjusted Life Years
RCT: Randomized Controlled Trial
