# Supplementary material for: Describing practices of priority setting and resource allocation in publicly funded health care systems of high-income countries
Source: BMC Health Serv Res. 2021 Jan 27;21:90. doi: 10.1186/s12913-021-06078-z (PMC7839200; doi:10.1186/s12913-021-06078-z)
Supplement: Supplementary file 1 — Additional file 1. [file 12913_2021_6078_MOESM1_ESM.docx]

**Supplementary Table 1 – PSRA initiatives identified in the study.**

|  | National level | State or Province / Regional level | Single health care organizations |
| --- | --- | --- | --- |
| Australia | HTA (PBAC & MSAC)  - No specific threshold | HTA (SAPACT) |  |
| Austria | Reimbursement decisions  - No single decision-making framework.  - For drugs, there is a legally defined process.  - Non- transparent decisions  Ad hoc decisions  - Loose reliance of several criteria | ------ | Hospital Clinical Committees  - Benefit assessment  - Budget impact |
| Canada | HTA of drugs (CADTH)  - “Overriding principle: will this work have an impact on the system?”  - Multi-criteria (including clinical burden, economic/financial impact and equity)  - Inputs from a variety of sources | Priorities and Evaluation Committee –PEC (a provincial institution)  - Single criterion: clinical effectiveness  - Decisions: high/medium/low priority for investment  - Some consideration of cost-effectiveness and budget impact without a formal influence on the final decision  Several health organizations have implemented formal processes based on A4R and/or PBMA. | Several health organizations have implemented formal processes based on A4R and/or PBMA |
| Denmark | Formal process for coverage of hospital and prescription drugs  - No further details | ------ | ------ |
| Finland | COHERE process  - Criteria:  - significance of a health issue  - medical justifiability  - ethical and economic considerations | ------ | ------ |
| France | National HTA  No other initiative known | ------ | ------ |
| Germany | No explicit PSRA processes  G-BA  - Reimbursement decisions for the statutory health insurance funds (GKV) | ------ | ------ |
| Netherlands | Zinnige Zorg (Appropriate Care)  - Systematic analyses per ICD-10 chapters  - Patient-oriented, effective and medically necessary care  HTA  - Consideration of multiple criteria, not only cost-effectiveness (e.g., budget impact, added therapeutic value)  MCDA / A4R  - Resource allocations at the Ministry of Health | ------ | ------ |
| New Zealand | No formal framework for resource allocation within the Ministry of Health  - historical allocation  - no explicit rationale  Decisions around package of services streamlined into the Ministry of Health.  - It specifies which services should be provided for the population  HTA (PHARMAC)  - Consideration of multiple factors situated within 4 dimensions: need, health benefits, costs and savings, and suitability. | ------ | ------ |
| Norway | National Guidelines on PS  - Produced/updated by the National Committee on Priority-Setting  - Approved by the parliament  - Three general criteria:  - benefit  - resource consumption  - severity  - No explicit process and much room for interpretation  - Despite important influence, there is evidence that it has not been used systematically  Nye Metoder  - Analysis for introduction of new technologies in specialized care  - HTA represents an important piece  - 3 types of HTA:  - Mini-HTA (hospital, supporting units)  - STA (single comparison)  - Full HTA (multiple comparisons performed at the national level) | ------ | ------ |
| Sweden | National ethical platform for PS  - 3 principles  - human dignity  - need-solidarity  - cost-effectiveness  - No explicit process  - Lots of room for interpretation/application  National guidelines on disease treatment  HTA  - Decentralized  - Separation between hospital drugs and prescription drugs | No single/unified formal framework  Several initiatives implemented and discontinued  Different strategies in place based on the national ethical platform  Ordered investments  - vertical and horizontal PS based on the national guidelines  HTA  - Several county councils have established a group to carry out mini-HTA |  |
| United Kingdom | HTA (NICE)  - Soft ICER threshold  - Well-described process in the literature  Advisory Committee on Resource Allocation – ACRA  - Criteria:  - equal access to care for equal need;  - reducing avoidable variations in health inequalities  Scottish Medicines Consortium  - ICER threshold with flexibility around drugs for end of life and rare diseases.  * Guernsey  PSRA framework: G1033  - 3 overarching notions:  - Allocative value  - Technical value  - Personal value  - Principles:  - maximization of value  - prioritization as the main tool for maximizing value  - cost-effectiveness  - proven clinical effectiveness  - work with the established budget  - equal treatment funding for equal needs  - no decision taken by third party on its behalf  - no discrimination in treatment  - Factors to be considered (among others):  - equity  - budget impact  - the anticipated outcomes of not investing in the proposal  - number of individuals affected  - NICE guidance is considered, but it is only advisory, not mandatory | Ad hoc decisions and historical allocations still seem to be the norm  No single framework for a given level of governance  CCGs  “Muddling through”  Multi criteria processes of variate designs  - Common criteria:  - strength of clinical effect  - strength of evidence/  - availability of alternatives  - fit with national and other guidance  - cost effectiveness | Ad hoc decisions and historical allocations still seem to be the norm |

**Supplementary Table 2 – Facilitators and barriers for the development and implementation of formal PSRA processes according to survey participants.**

| Country | Facilitators | Barriers |
| --- | --- | --- |
| Australia | - Strong leadership  - HTA capacity  - Political understanding of the importance of a formal process | - Fragmentation of the health care system  - Lack of trust among stakeholders (especially on the clinicians’ side)  - Lack of transparency on evaluation processes |
| Austria | - Budgetary pressure (overpriced drugs)  - Courage to stand up against pressure from clinicians and industry  - Existence of champions in key organizations | - Incentives to work too closely with industry  - Lack of precise definition of ‘innovation’ within the medical culture  - Lack of a culture of transparency  - Hierarchical structures and MD dominance |
| Canada  (CADTH) | - Nimble culture  - Service orientation | - Vested interests  - Short-term perspective  - Discomfort in weighting criteria |
| Canada (provincial institution) | - Strong leadership  - Budgetary pressures (overpriced drugs & rise in number of cases)  - Genuine commitment among committee members to achieve the best outcomes  - Perceived legitimacy | - Lack of knowledge on PSRA frameworks  - Unwillingness to consider other criteria, apart from clinical effectiveness  - Discontinuity of high level personnel  - Lack of administrative support  - Lack of dedicated human resources  - Resistance from clinicians and politicians  - Lack of public education on cancer and PSRA |
| Denmark | ----- | - Lack of knowledge on PSRA |
| Finland | ----- | - Fragmentation  - Lack of funding to establish formal processes |
| France | - Strong leadership | - Reluctance to change  - Financial disincentives  - Little development of research on PSRA |
| Germany | ----- | - Politicians resistance (afraid of electoral consequences)  - Resistance from clinicians |
| Netherlands | - Learning culture  - Integrity  - Culture of Transparency  - Stakeholders engagement | - Vested interests  - Lack of trust among stakeholders  - Black box procedures |
| New Zealand | - Desire for better performance and less variation  - Excellence recognition of the HTA agency (PHARMAC) | - Too many players and too many levels  - Conflict of interests  - Culture of secret negotiations (assuming better deals)  - Misunderstanding of PSRA and its real causes |
| Norway | - Open discussion  - Presence of key champions  - Good understanding of the topic by politicians  - Strong involvement of research expertise  - Strong welfare system that emphasizes equity  - Highly educated population with strong tradition of fair political decisions | - Lack of precise PSRA guidance beyond general principles  - Lack of trust between stakeholders  - Resistance to change  - Vested interests  - Culture of secret price negotiations  - Discrepancy between who makes decision and who holds the budget in terms of drugs |
| Sweden | - Strong leadership at all levels  - Good dialogue between politicians, administrators and health care professionals at the national level  - Openness to change  - Sense of responsibility for the entire health system  - Good adherence to decisions | - Lack of trust among stakeholders at the regional level  - Lack of adequate understanding of PSRA  - Political resistance to communicate difficult topics  - Reliance on historical allocations  - Emphasis on adoption  - Vague provision of principles  - Media pressure |
| Switzerland | ----- | - Lack of a culture of transparency |
| United Kingdom  (national level) | - Good data management in health system  - Budgetary pressures  - Strong leadership  - Strong emphasis on performance improvement  - Fear of the consequences of unplanned cuts  - Independent nature of NICE  - Great support of the research community | - Strong backlash of negative recommendations  - Political interference  - Media pressure  - Vested interests  - Lack of time to engage in new activities (lack of personnel)  - Costs of stakeholder involvement  - Avoidance of tough decisions  - Unwillingness to be open and transparent (NHS Dentistry)  - Perceived tension between what organization is expected to do nationally….and what local people may want - so they don't engage (risk is perceived to be too high to organization). |
| United Kingdom (local/regional level) | - Budgetary pressures (economic downturn)  - ‘Air cover’ from NICE  - Sharing of resources at local/regional levels  - Environment that foster discussion of PSRA in the health care system | - Political toxicity of any suggestion of rationing  - Lack of funding in local evidence generating and capacity building  - Lack of consistency  - Constant reorganization  - Discontinuity of personnel (commissioners) |

**Supplementary Table 3 – Strengths and areas for improvement in the current practices according to survey participants.**

| Country | Strengths | Areas to improve |
| --- | --- | --- |
| Australia  (HTA) | - Rigor and consistency in appraisal  - Procedural fairness  - High level of expertise | - Decision-making remains flexible in relation to the advice emerging from evaluation  - “Greater capacity to assess technologies in the context of the whole system”  - Lack of personnel “on the ground”, outside academia |
| Austria | ------ | - Increasing transparency  - Standardization of processes  - Collaborative priority setting across sectors  - Expand PSRA beyond acute or end-of-life care |
| Canada (CADTH) | - Criteria based  - Evidence informed | - Transparency (topics, criteria and influencing actors)  - Improving understanding of all involved that resources are limited and choices need to be made. |
| Canada (provincial institution) | - Formal committee  - Good buy-in/adherence from/by clinicians  - Rigorous evaluation of the clinical evidence  - Quick turn-around time  - Interval review (higher trust) | - Expansion of focus (use of multiple criteria and other types of evidence)  - Transparency  - Improving knowledge of committee members on PSRA  - Patient participation  - Public engagement  - Inclusion of ethicists and health economists  - Appeals mechanism |
| Denmark | - Good approach to budgeting at regional and national levels  - Reasonably knowledgeable politicians | - Systematic use of HTA and CEA/CUA |
| Finland | ----- | - Legislation  - Funding  - Staffing |
| France  (HTA) | - Publicity of HTA decisions | - More research  - Timeline Incongruence  (technologies can be disseminated based upon political decision before the formal assessment is finalized) |
| Germany | ---- | ----- |
| Netherlands | - Transparent process  - Use of deliberative MCDA  - Continuous process improvement  - Skilled personnel | - Lack of guidance on interpretation of criteria  - No clear link with health system values  - Stakeholder involvement |
| New Zealand | - Good HTA process (PHARMAC) | - Poor PSRA practices within the Ministry of Health  (outside PHARMAC in general)  - Transparency / clarity of the decision-making process (how decisions are made, how criteria are weighted, who influence the process  - Capacity building  - Consumer involvement  - Auditing processes |
| Norway | - Legally defined principles  - Political legitimacy  - Fairness  - Explicit talk | - Better use of evidence  - Stronger follow-up  - Transparency  - Stakeholder involvement  - More awareness on PSRA among stakeholders and students  - Speed  - Public engagement  - Disinvestment |
| Sweden | - Transparency (National level)  - Explicit ethical platform  - Democratic composition of New Therapy Councils (HTA) | - Capacity building:  a) Establish a common view on why and how more formal priority setting matters  b) how to distribute and define different roles between actor  c) how to improve the trust between the key actors  d) to create a sense of mutual responsibility for a universal healthcare system  (This internal foundation for PSRA is necessary to engage in formal processes to secure legitimacy within the whole society)  - Integration of PSRA with budget establishment in the health authorities (move beyond the common-sense view that PS is a ‘special activity’, risky and difficult)  - Transparency (county/regional level)  - Translation of the ethical platform in concrete practices  - Focus on adoption of new drugs  - Disinvestment  - More focus on opportunity cost  - Stakeholder involvement  - Better distinction between hospital drugs and prescription drugs |
| Switzerland | ----- | - Transparency / clarity of decision-making processes  - Less power of lobbyists  - More openness to discuss priorities at the local level |
| United Kingdom  (NICE) | - Accountability  - Clear principles  - Transparency  - “A track record of learning from mistakes and from a wide range of perspectives”  - “Clear distinction between scientific evidence and policy preferences”  - Broad spectrum in stakeholder involvement  - Infrastructure  - High level of public scrutiny | - Consideration of social values  - Public engagement  - Better understanding of health care costs and trade-offs  - Make more explicit the final decision-making process  - Criteria weighting (consistency among decisions) |
| United Kingdom  (regional – CCGs) | - Stakeholder involvement  - Flexibility  - Knowledge of local reality  - Good dialogue both ways between commissioners (the decision-makers) and NHS contract holders | - Clarity and explicitness  - Consistency /Continued and systematic processes  - Clinicians need more confidence to be open with patients about all relevant factors in decision-making at the consultation level.  - Public engagement and education |
| United States | ----- | ----- |
