# Supplementary material for: Describing practices of priority setting and resource allocation in publicly funded health care systems of high-income countries
Source: BMC Health Serv Res. 2021 Jan 27;21:90. doi: 10.1186/s12913-021-06078-z (PMC7839200; doi:10.1186/s12913-021-06078-z)
Supplement: Supplementary file 2 — Additional file 2. Questionnaire [file 12913_2021_6078_MOESM2_ESM.docx]

**Additional file 2 – Questionnaire**

Personal Information

- Name: ______________________________________________________________
- Country (current place of work): ___________________________________________
- Email address: _________________________________________________________

1) Which job title best designates your main role?

- Decision-maker
- Academic / Researcher

2) What is the organizational context of priority-setting within the health care system you are most familiar with?


a) In terms of level of governance:

- National level
- State or Provincial level
- Regional level (e.g., health authority, health district, health region)
- Single organizational level (e.g., hospital, community organization, residential care facility)
- Other. Please specify: ________________________________________

b) And in terms of scope or context? (Check ALL that apply.)

- Drugs
- Non-drug health technologies
- Work force / Human resources
- Specific fields of care (e.g., primary care, cancer, public health). Please name them: ____________________________________________________
- Other(s). Please specify: ___________________________________________

3) How does the organization you are most familiar with establish priorities to invest in? Is there any formal process or framework that is used for that purpose? What is the underlying rationale or principles in decision making (even if there is no formal process)?

________________________________________________________________

________________________________________________________________

________________________________________________________________

________________________________________________________________

________________________________________________________________

4) Conversely, how are priorities established for disinvestment? Is there a formal process for that purpose and if so, is it linked to the investment priority setting process? On what basis/rationale are decisions for disinvestment made?

________________________________________________________________

________________________________________________________________

________________________________________________________________

________________________________________________________________

________________________________________________________________

5.a) Which stakeholders are commonly involved in the process of decision-making in priority-setting?

Check ALL that apply.

- Politicians
- Managers / Administrators
- Health professionals
- Patients
- Other(s). Please specify: _____________________________________

5.b) How are these stakeholders involved? How is their input incorporated into the process of decision-making?

________________________________________________________________

________________________________________________________________

________________________________________________________________

________________________________________________________________

________________________________________________________________

6) Does the general public participate in the process in any way? If so, is there direct involvement somehow or are there specific strategies to elicit public preferences?

________________________________________________________________

________________________________________________________________

________________________________________________________________

________________________________________________________________

________________________________________________________________

7.a) What types of evidence/information are taken into account? 
 
Check ALL that apply.

- Epidemiological evidence
- Clinical evidence
- Economic evidence
- Expert opinions
- Other(s). Please specify: ________________________________________

b) And how specifically is evidence integrated into the process of decision-making?

________________________________________________________________

________________________________________________________________

________________________________________________________________

________________________________________________________________

8) What are the facilitators for the development and implementation of formal and explicit processes of priority-setting you can identify in the context you are involved with or study (e.g., strong leadership, culture to learn, openness to change, etc.)?

________________________________________________________________

________________________________________________________________

________________________________________________________________

________________________________________________________________

9) What are the barriers for the development and implementation of formal and explicit processes of priority-setting you can identify in the context you are involved or study (e.g., discontinuity of personnel, misalignment of incentives, lack of trust between stakeholders, etc.)?

________________________________________________________________

________________________________________________________________

________________________________________________________________

________________________________________________________________

10) Is there any mechanism for appealing decisions once made?

- Yes
- No
- Uncertain

10.a) If yes, please explain this appeals process:

________________________________________________________________

________________________________________________________________

________________________________________________________________

________________________________________________________________

11) Are the current practices perceived to be fair?

|  | Yes | No | Prefer not to answer | Do not know |
| --- | --- | --- | --- | --- |
| By involved stakeholders |  |  |  |  |
| By the general public |  |  |  |  |
| In your opinion |  |  |  |  |

12) Overall, how would you rate the current priority-setting process in terms of performance?

|  | Very poor | Poor | Fair | Good | Very good |
| --- | --- | --- | --- | --- | --- |
| Overall performance |  |  |  |  |  |

13) In your view, what are the main strengths within the current priority setting processes?

________________________________________________________________

________________________________________________________________

________________________________________________________________

________________________________________________________________

14) What specific areas could be improved within the current priority setting processes?

________________________________________________________________

________________________________________________________________

________________________________________________________________

________________________________________________________________

Do you know other researchers or decision-makers in your country that may also be able to contribute to this project?  Could you please list below their names and institutional affiliation (and/or email address), as long as this is publicly available information? Otherwise, please feel free to share the survey link with these potential participants.

- 1. ________________________________________________
- 2. ________________________________________________
- 3. ________________________________________________
- 4. ________________________________________________
- 5. ________________________________________________
